# Vehicle Collision Prediction under Reduced Visibility Conditions

**DOI:** 10.3390/s18093026

**Published:** 2018-09-10

**Authors:** Keng-Pin Chen, Pao-Ann Hsiung

**Affiliations:** Department of Computer Science & Information Engineering, National Chung Cheng University, Chiayi 62102, Taiwan; go120625@gmail.com

**Keywords:** vehicle collision avoidance, data analytics, prediction, time-to-collision, back-propagation neural network, data fitting

## Abstract

Rear-end collisions often cause serious traffic accidents. Conventionally, in intelligent transportation systems (ITS), radar collision warning methods are highly accurate in determining the inter-vehicle distance via detecting the rear-end of a vehicle; however, in poor weather conditions such as fog, rain, or snow, the accuracy is significantly affected. In recent years, the advent of Vehicle to Vehicle (V2V) and Vehicle to Infrastructure (V2I) communication systems has introduced new methods for solving the rear-end collision problem. Nevertheless, there is still much left for improvement. For instance, weather conditions have an impact on human-related factors such as response time. To address the issue of collision detection under low visibility conditions, we propose a Visibility-based Collision Warning System (ViCoWS) design that includes four models for prediction horizon estimation, velocity prediction, headway distance prediction, and rear-end collision warning. Based on the history of velocity data, future velocity volumes are predicted. Then, the prediction horizon (number of future time slots to consider) is estimated corresponding to different weather conditions. ViCoWs can respond in real-time to weather conditions with correct collision avoidance warnings. Experiment results show that the mean absolute percentage error of our velocity prediction model is less than 11%. For non-congested traffic under heavy fog (very low visibility of 120 m), ViCoWS warns a driver by as much as 4.5 s prior to a possible future collision. If the fog is medium with a low visibility of 160 m, ViCoWs can give warnings by about 2.1 s prior to a possible future collision. In contrast, the Forward Collision Probability Index (FCPI) method gives warnings by only about 0.6 s before a future collision. For congested traffic under low visibility conditions, ViCoWS can warn a driver by about 1.9 s prior to a possible future collision. In this case, the FCPI method gives 1.2 s for the driver to react before collision.

## 1. Introduction

Data collected by the US National Highway Traffic Safety Administration have shown that rear-end collisions constitute the highest percentage of collisions of motor vehicles in transport, and rear-end collisions often lead to multiple injuries and property damage [1]. To address the rear-end collision problem, Collision Warning Systems (CWS), such as radar systems that detect and warn drivers about potential rear-end collisions, are often used [2]. Radar systems are highly accurate in determining the inter-vehicle distances via detecting the rear-end of vehicles; however, under poor weather conditions such as fog, rain, or snow, its accuracy is significantly affected. Rasshofer et al. showed that the transmit power density of laser radar is large, and “the high reflectivity of snowflakes generates a false alarm rate of up to 2.5/s·m^2^ in a range of 5 m in front of the sensor” [3].

In the early 2000s, the study of wireless network for data exchange in vehicular environments became popular in the field of Intelligent Transportation Systems (ITS). A commonly used technology in ITS is the dedicated short-range communication (DSRC), which is a wireless communication technology for Vehicle-to-Vehicle (V2V) or Vehicle-to-Infrastructure (V2I) communication, compliant with the IEEE 802.11p (physical layer protocol) standard and the IEEE 1609 (network layer protocol) standard [4]. DSRC-based collision warning systems have been proposed to reduce the number of accidents under poor weather conditions [5].

Although DSRC-based systems, similar to radar-based systems, allow communication among vehicles and warn drivers in real-time, low visibility conditions are still a major concern. Weather conditions have an impact on human-related factors such as response time. Further, though human factors significantly impact collision estimations; however, most collision warning methods do not consider human factors adequately [6], thus resulting in high rates of false alarms or missing alarms.

In this work, our goal is to design a system that can not only predict vehicle collisions, but also dynamically lengthen the prediction horizon (number of future time slots for prediction) such that the system can enhance safety through providing early warnings and giving drivers enough time to react to future possible collisions.

This article is organized as follows: Section 2 presents related work on collision warning models, human factors, and vehicle speed prediction. Section 3 presents the proposed Visibility-based Collision Warning System design. Section 4 gives the experiment results. Section 5 concludes the article with some future work.

## 2. Related Work

Car following models are the basis of microscopic simulation models, and are often used to analyze vehicle behavior such as following distances and the speed at which a vehicle moves in different time slots. A car following model controls a driver’s behavior with respect to the preceding vehicle in the same lane [7]. In this work, we build a collision warning model based on the car following scenario. Rear-end collision warning models can be classified into two categories, namely parametric models and non-parametric models. They are introduced and compared as follows.

### 2.1. Research on Collision Warning Models

In parametric models, the Time-To-Collision (TTC) model is one of the most common models. Hayward [8] defined TTC as “the time required for two vehicles to collide if they continue at their present speeds and on the same path”. The TTC model is computed as shown below:

TTC = *D*/Δ*V* (1)

where *D* is the distance between the center of a subject vehicle (SV) to the center of the lead vehicle (LV) and Δ*V* is the relative speed. However, there are some limitations in the TTC model. First, it cannot be computed when the relative speed is negative or zero. Second, several modified TTC algorithms have been proposed, but they do not suggest the best timing for warning because they do not consider human factors under different traffic conditions [6]. The stopping distance algorithm (SDA) with kinematics analysis is one of the most common models. An et al. [9] proposed the SDA model for 3 scenarios, namely lead vehicle stopped (LVS), lead vehicle decelerating (LVD), and lead vehicle moving (LVM). In LVS and LVM scenarios, the SDA model is computed as follows:

(2) $$D_{W}=\frac{{\left(V_{F}-V_{L}\right)}^{2}}{-2a_{F}}+\left(V_{F}-V_{L}\right)\times \left(T_{R}+t_{sys}\right)+D_{S}$$

In a LVD scenario, the SDA model is computed as follows:

(3) $$\;D_{W}=\frac{V_{F}^{2}}{-2a_{F}}+V_{F}\times \left(T_{R}+t_{sys}\right)-\frac{{\left(V_{L}\right)}^{2}}{2a_{L}}+D_{S}$$

where *V_F_* and *V_L_* are the velocities of the following vehicle and the lead vehicle, respectively, *T_R_* is the reaction time of a driver in the vehicle, and *a_F_* and *a_L_* are the braking intensities of the two vehicles, respectively, *t_sys_* is system delay, and *D_S_* is a desired safety gap when the velocity of the following vehicle is the same as that of the lead vehicle. Xiang et al. introduced a vehicle kinematics (VK) model, based on the DSRC technology. The authors proposed the model to solve information delay and transmission delay from DSRC. However, problems still exist in the kinematics rear-end CW model, that is, either high false alert or high missing alert occurs in the model due to the delay [10].

**Figure 1:**
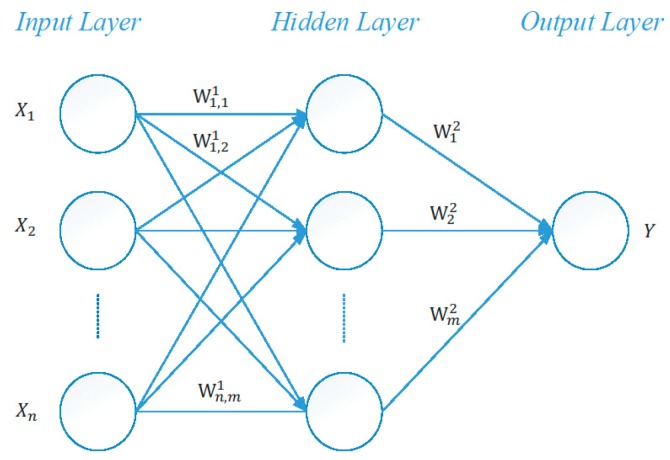
A typical three-layer back-propagation neural network.

In non-parametric models, artificial neural networks (ANN) are one of the most common models. Figure 1 shows a basic architecture of a multi-layer back-propagation neural network (BPNN). Xiang et al. used a neural network model to address the problem of high frequency of false alerts and missing alerts occurring in the VK model [10]. Lee et al. also proposed a framework for real-time CWS based on a Multi-Layer Perceptron Neural Network (MLPNN) in V2I environment [6]. The authors tried to solve the impact of different driver reaction times in a rear-end CWS. But a case study done in St. Petersburg, Russia in 2014 pointed out that the costs of V2I technology are very high due to network deployment of Road Side Units (RSU). It is more feasible to use DSRC for V2V applications [11]. An et al. used fuzzy logic for coarse assessment, and the ratio of collision occurrence for fine assessment. Then, the authors used the neural network ensembles to calculate collision probabilities [12]. Wu et al. proposed a Crash Risk Increase Indicator (CRII) for traffic in fog conditions. It included not only local traffic flow characteristics but also traffic flow relationships. It was shown using a logistic model that crash risk is prone to increase at ramp vicinities and locations with heavy traffic in fog conditions [13]. Wu et al. also proposed a Rear-End Collision Risk Index (RCRI) which used the safe stopping distances of two consecutive vehicles in the same lane and the clearance distance between them (so that they do not collide into each other). Different vehicle types, different lanes, and driving maneuver can affect the accuracy of RCRI [14].

### 2.2. Research on Human Factors

Two human factors can be considered for traffic safety namely the perception-reaction time (PRT) and the visibility due to weather conditions. They are introduced and compared as follows.

PRT or “break reaction time” is an important human factor, which changes under complex traffic conditions. Layton et al. stated that “the perception-reaction time for a driver is often broken down into the four components that are assumed to make up the perception reaction time. These are referred to as the PIEV time or process”. PIEV is short for perception, intellection, emotion, and volition [15], which are defined as follows:

- Perception is “the time to see or discern an object or event”.
- Intellection is “the time to understand the implications of the object’s presence or event”.
- Emotion is “the time to decide how to react”.
- Volition is “the time to initiate the action, for example, the time to engage the brakes”.

In 1998, Seiler et al. compared Mazda’s algorithm and Honda’s algorithm for braking critical distance, then the authors proposed a modified algorithm which considered human factors [16]. In 2010, Chang et al. designed a three layer back-propagation neural network to evaluate the PRT from sensing data [17]. In 2012, Kusano et al. proposed the probability density function of PRT, and classified it into three categories, namely fast, medium, and slow [18]. In 2015, Halmaoui et al. proposed a quantitative model to describe the relationship between visibility and PRT during daytime fog. Then, the dehazing of images was performed using visibility enhancement algorithms in a Head Up Display [19].

Visibility has two definitions. One is the meteorological visibility: “the distance at which the contrast of a black object is attenuated by 95%”, and the other one is the target visibility: “it rates to what extent a target is detected in a scene, when an observer looks at it” [19]. Meteorological visibility can be estimated by detection of atmospheric light extinction from weather stations or visibility cameras, and the range is in kilometer level [20]. Target visibility can be estimated by camera equipped in a vehicle, and the range is in meter level [21]. In this work, we use target visibility, because the information for meteorological visibility cannot represent visibility on the reads if the weather stations are not close enough to traffic stations [22]. Van Der Horst et al. analyzed the relationship between visibility and driver behavior using inductive loop data, then found that driver speeds are much lower in fog than in clear visibility, but following distances decrease in fog. Especially in the visibility range between 40 m and 120 m, free-driving speeds are too high to allow for a successful stop for a stationary object [23]. Halmaoui et al. also found the relationship between visibility and PRT during daytime fog [19]. Most work focus on how to detect and enhance vision under low visibility, such as in fog [24]. However, we do not speculate on how to convert foggy images into clear ones for some target visibility. This work focuses on “how to improve performance of rear-end CWS under poor visibility conditions.” Based on the research work mentioned above (mainly [19]), it is concluded that target visibility can affect driver speed, following distances, and the PRT.

### 2.3. Research on Vehicle Speed Prediction

Park et al. presented a Neural Network Traffic Modeling-Speed Prediction (NNTM-SP) algorithm to predict traffic speed profile based on real time traffic data. It can predict speed profile in the future for up to 30 min [25]. Lefèvre et al. [26] compared six models: Constant Speed model (CS), Constant Acceleration model (CA), SUMO model, Intelligent Driver Model (IDM), Gaussian Mixture Regression model (GMR), and Artificial Neural Network model (ANN) for predicting the ego-vehicle’s speed on a highway. In the case of long-term predictions, the ANN model performed better than the others. The results show that the non-parametric approaches, such as GMR and ANN models, were better than the parametric models for all the tested prediction horizons. Jing et al. [27] proposed a method to predict speed in Cooperative Adaptive Cruise Control (CACC) using V2V communication. The general idea of the speed prediction algorithm is to estimate the propagation characteristics of speed perturbations from the lead vehicle to the preceding vehicle for a given convoy, so that the preceding vehicle’s speed changes corresponding to a given perturbation can be calculated as soon as it acts on the lead vehicle. Jiang et al. proposed a two-level prediction method based on the NN model and the Hidden Markov model (HMM) for vehicle speed prediction, for which the 98.7th percentile absolute error was 1 m/s [28].

## 3. Visibility-Based Collision Warning System Design

The target problem in this work is to design a system that can not only predict vehicle collisions, but also enhance the safety of driving by giving early warnings. In prediction systems, there is often a tradeoff between how far into the future you can predict accurately and how far into the future the prediction is actually required for the target application. For example, the average prediction errors in a smart grid system go from 4.58% in the first time slot to 9.67% in the second time slot, and then 12.61%, 14.89%, and 16.32% in the 3rd to 5th time slots, respectively. If only 15% error is tolerable, then the only 4 time slot predictions can be considered; however, for proper smart grid operations, predictions for wind speed, irradiance, and load consumption in the future 5 or more time slots may be desirable.

**Figure 2:**
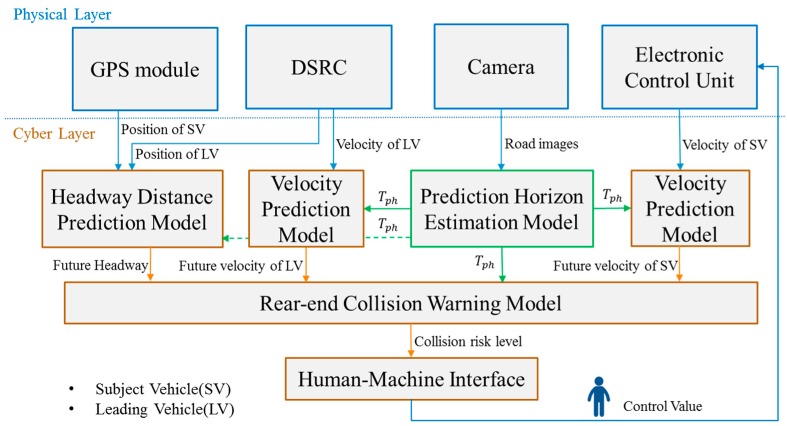
Visibility-based Collision Warning System Design.

**Figure 3:**
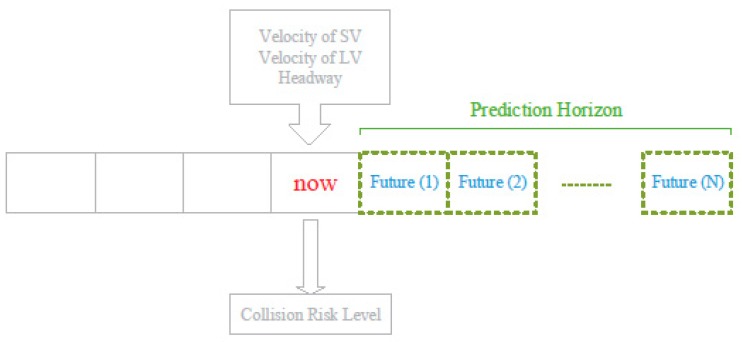
Core Concept in Visibility-based Collision Warning System (ViCoWS).

To solve the above target problem, we propose a Cyber-Physical System (CPS) called Visibility-based Collision Warning System (ViCoWS), whose architecture is illustrated in Figure 2. ViCoWS consists of a physical part and a cyber part. The physical part consists of GPS module, DSRC module, camera, and Electronic Control Unit (ECU). The cyber part consists of 4 types of models, including headway distance prediction model, velocity prediction models (one for Subject Vehicle (SV) and one for Leading Vehicle (LV)), prediction horizon estimation model, and rear-end collision warning model. The future velocities of SV and LV are predicted by the velocity prediction models and the future headway distance between SV and LV is predicted by the headway distance prediction model. Using the two series of future velocities of SV and LV and the headway distances in all time slots in the prediction horizon, a collision risk level is predicted as shown in Figure 3, which uses TTC as defined in Equation (1). Note that the prediction horizon is not fixed in this system, rather it is dynamically changed using the prediction horizon estimation model. For changing the prediction horizon, this model takes human factors into consideration by estimating PRT under different visibility conditions [19]. Under severe visibility conditions, PRT will be estimated to be longer than normal, and thus the prediction horizon will be increased so as to be able to predict future possible collisions and thus enhance driving safety. The different models will be discussed in details in the rest of this section. When the collision risk level is high, the human-machine interface (HMI) will warn the driver.

### 3.1. Prediction Horizon Estimation Model

Halmaoui et al. proposed a quantitative model to estimate the PRT under a target visibility [19]. When target visibility becomes low, driver’s PRT is expected to become long. Some limits exist in the quantitative model. First, the experiments did not consider the data with detection rate below 50%. A 50% detection rate means the target has 50% chance to be seen when we look at it. Second, PRT longer than 2.5 s were not considered. With a camera sensor, the system monitors the environment in real time. Thus, target visibility can be computed via visibility distance estimation employed in image processing model [21]. Then, the quantitative model computes driver’s PRT under the current target visibility.

Given the estimated PRT, our proposed prediction horizon estimation model transforms the PRT into a prediction horizon for velocity prediction, as follows:

(4) $$T_{ph}=\{\begin{array}{c} 0.932T_{pr}^{3}-4.6822T_{pr}^{2}+10.48T_{pr}+13.16\;\mathrm{if}\;V_{LV}\geq 30\\ -0.0207T_{pr}^{3}+0.3642T_{pr}^{2}+0.2078T_{pr}+0.6447\;\mathrm{if}\;V_{LV}<30 \end{array}$$

where *T_ph_* is the prediction horizon, *T_pr_* is the SV driver’s PRT in seconds, and *V_LV_* is the LV speed in feets per second. A third order polynomial quantitative function is used to fit the data. The round function is used to make *T_ph_* an integer. It can be observed that the longer the PRT is, the longer the prediction horizon is.

### 3.2. Velocity Prediction Model

Velocities for SV and LV are both predicted using a back propagation neural network (BPNN), which consists of 3 layers namely input, hidden, and output. Past velocities in the time slots *t* − *n* to *t* (current time slot) are used as input data for training the model so as to predict the velocities in the future time slot *t +* 1. The output layer uses hyperbolic tangent sigmoid transfer function. The training samples is about 125,260 and the velocity range is from 0 ft to 100 ft.

### 3.3. Headway Distance Prediction Model

The headway distance between SV and LV are estimated or predicted as follows:

$$h=H+D_{err}+T_{d}\left(V_{SV}-V_{LV}\right)$$

where *H* is the headway distance, *D_err_* is the GPS positioning error, *T_d_* is the information delay which includes GPS delay, transmission delay, and computation time of collision detection, *V_SV_* and *V_LV_* are the velocities of SV and LV, respectively. The future headway distance is computed as follows:

$$D\left(t+1\right)=D\left(t\right)+\frac{V_{LV}\left(t+1\right)-V_{SV}\left(t+1\right)}{p}$$

where *D*(*t*) is the space headway in the time slot *t* and *t* + 1, respectively, $V_{LV}\left(t+1\right),\;V_{SV}\left(t+1\right)$ are the predicted velocities of LV and SV for the time slot *t* + 1, respectively, *p* is the period of one time slot, which is 0.1 s in this work.

### 3.4. Rear-End Collision Warning Model

**Figure 4:**
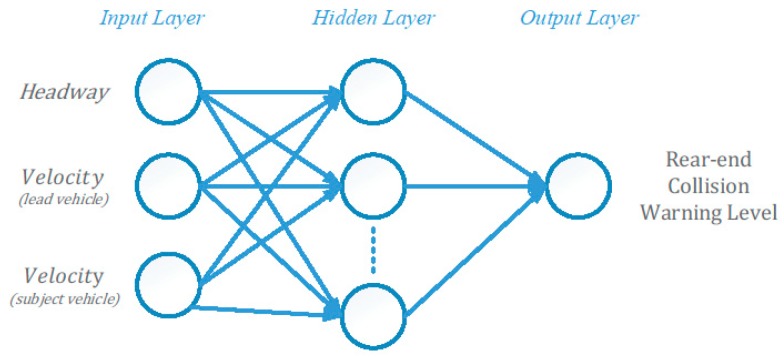
Rear-end Collision Warning based on BPNN Model.

As shown in Figure 4, the rear-end collision warning model is based on BPNN. It consists of 3 layers including input layer, hidden layer, and output layer. There are three input neurons in the input layer, including the headway distance (*h*) between SV and LV, the LV velocity (*V_LV_*), and the SV velocity (*V_SV_*). The number of nodes in the hidden layer is twice as much as that in the input layer. We adopt the Levenberg-Marquardt method to train the BPNN and select hyperbolic tangent sigmoid transfer function for neuron activation. First, we calculated TTC as defined in Equation (1) with real-time data, and transformed it into a collision warning level using a Forward Collision Probability Index (FCPI) [29]. Then, we trained the network to make the output represent the collision warning level, which is a ratio between 0 and 1. Finally, we predicted the collision warning levels for all time slots in the prediction horizon. The final collision warning level was computed by using the maximum function over all predicted warning levels in the prediction horizon.

FCPI used a Z-shaped membership function to estimate collision probability as shown in Equation (5):

(5) $$f\left(x,a,b\right)=\{\;\begin{array}{c} 1,\;\mathrm{if}\;x\leq a\\ 1-2{\left(\frac{x-a}{b-a}\right)}^{2},\;\mathrm{if}\;a\leq x\leq \frac{a+b}{2}\\ \begin{array}{c} 2{\left(\frac{x-b}{b-a}\right)}^{2},\;\mathrm{if}\;\frac{a+b}{2}\leq x\leq b\\ 0,\;\mathrm{if}\;x\geq b \end{array} \end{array}$$

where the parameters *a* and *b* are set as 0.5 and 2.5, respectively, according to the three warning levels of TTC: “overriding (TTC < 0.5 s)”, “Imminent warning (0.5 s ≤ TTC < 1.5 s)”, and “cautionary warning (1.5 s ≤ TTC < 2.5 s)” [30]. If TTC is less than 0.5 s, the probability of collision is 100%. If TTC is larger than 2.5 s, the probability of collision is 0%. The probability of collision will increase quickly when TTC is between 0.5 s and 1.5 s, and the probability of collision will increase slowly when the TTC is between 1.5 s and 2.5 s. It is suggested that the collision warning level threshold be set as 0.5, at which the system should warn a driver to slow down [6]. Training data are collected from the Next Generation Simulation (NGSIM) US Route 101 Dataset [31].

## 4. Experiments

In this section, we present the experimental results of the proposed ViCoWS system. We first introduce the experimental environment and test data samples. Next, we determine the parameter settings for the proposed method and also give velocity prediction results and collision warning level prediction results. The experimental environment used in this work is as follows. We used a PC with an Intel(R) Core(TM) i7-4790 CPU having four cores and the frequency is 3.60 GHz. There are 16 GB memory. We used 64-bits Windows 10 as our operating system. Our programs were written in the tool Matlab R2016a.

### 4.1. Test Data

The Next Generation Simulation (NGSIM) US Route 101 Dataset was used [31]. It was collected from a segment of U.S. Highway 101 in Los Angeles, California, in June 2005. It is provided by tracking individual vehicular movements every 0.1 s, including vehicle location, length, width, velocity, acceleration, the preceding vehicle id, the following vehicle id, space headway, and time headway.

We tested the model with two special segments of the vehicle data. One is non-congested traffic scenario (LV’s speed ≥ 30 ft/s) and the other is congested traffic scenario (LV’s speed < 30 ft/s).

**Figure 5:**
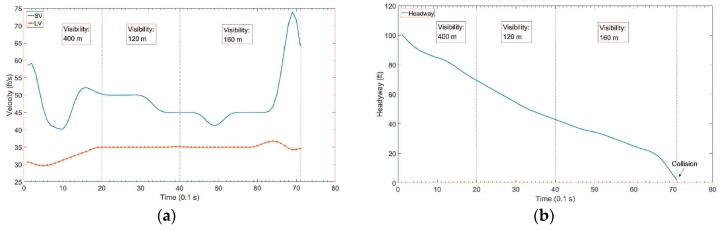
Test data for non-congested traffic scenario. (**a**) Velocity Profile; (**b**) Headway Profile.

For the non-congested traffic scenario, the velocity profile and the headway profile are shown in Figure 5. Both vehicles SV and LV were in the same lane before the 71st time slot. The SV speeded up at the 10th time slot, 49th time slot, and 62nd time slot. After the 71st time slot, SV passed the LV. We assume that there was a car accident between SV and LV at the 71st time slot, if SV did not to change the lane when the headway was close to zero. For target visibility, the following assumptions were made. From the 1st to 19th time slots, the target visibility is 400 m. From the 20th to 39th time slots, the target visibility is 120 m due to a heavy fog. From the 40th to 71st time slots, the target visibility is 160 m due to a medium fog. The target visibility was basically assumed; however, its validity was confirmed through weather experts.

**Figure 6:**
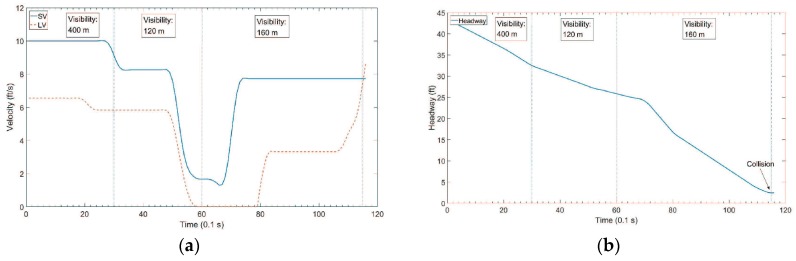
Test data for congested traffic scenario. (**a**) Velocity Profile; (**b**) Headway Profile.

For the congested traffic scenario, the velocity profile and the headway profile are shown in Figure 6. The SV and LV were in the same lane in all time slots. The LV decreased its speed to zero from the 45th time slot to the 79th time slot, and speeded up at the 80th time slot. We assume that there was a car accident between SV and LV at the 115th time slot, because the headway was close to zero. For target visibility, the following assumptions were made. From the 1st to 39th time slots, the target visibility is 400 m. From the 40th to 79th time slots, the target visibility is 120 m due to a heavy fog. From the 80th to 115th time slots, the target visibility is 160 m due to a medium fog.

### 4.2. Experiment Results

We give the experiment results for our proposed method, including deciding the number of training inputs for the model, velocity prediction, and the time series of collision warning levels for the test data. For training purposes, we used 125,260 velocity data of the vehicles and we used 437 velocity data for testing the accuracy of the trained model for velocity predictions. The training data were divided into three ranges of velocity 0 to 20 ft, 20 to 60 ft, and 60 to 100 ft, so that we have a more complete coverage of possibilities velocities for training.

#### 4.2.1. Deciding the Number of Training Inputs for Velocity Prediction Model

We used the velocity information to train the BPNN-based velocity prediction model. The number of training inputs means the number of time slots considered when training the BPNN-based velocity prediction model. For example, if the sampling rate is one velocity data sample per 0.1 s, then using three training inputs means taking previous 0.3 s velocity data to train the model. The goal of this experiment is to determine a suitable the number of input nodes for the BPNN model.

We calculate the error with mean squared error (MSE), as shown in Equation (6), which is used to estimate the error for training:

(6) $$\mathrm{MSE}=\frac{1}{n}\overset{n}{\underset{i=1}{\sum }}{\left(\frac{A_{i}-P_{i}}{A_{i}}\right)}^{2}$$

where *n* is the number of training samples, *A_i_* is the actual velocity of *i*th training sample, and *P_i_* is the predicted velocity of the *i*th training sample.

**Figure 7:**
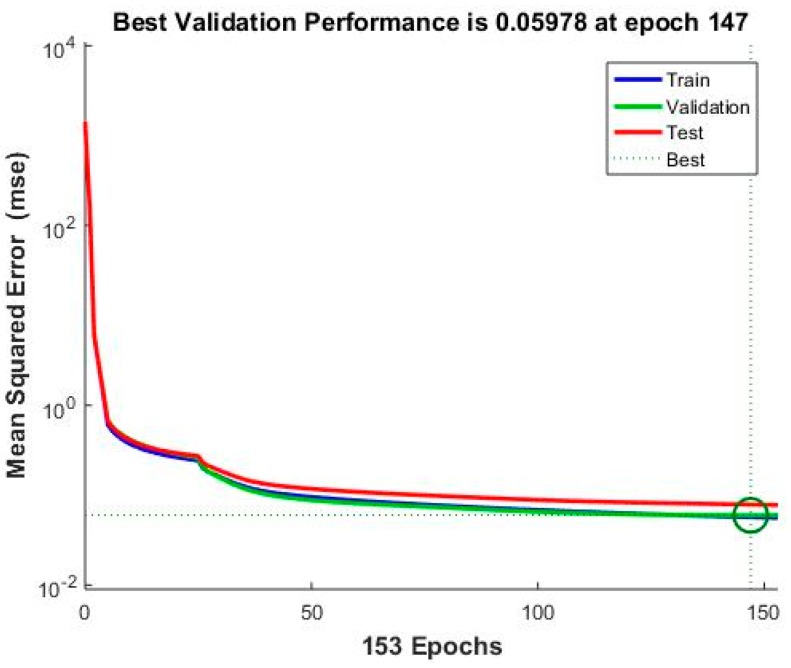
Performance of velocity prediction based on BPNN model with 8 input nodes.

The error reduces after more epochs of training, but might start to increase on the validation data set when the network starts overfitting the training data. The best performance is taken from the epoch with the lowest validation error. For example, Figure 7 shows the performance of the BPNN model with eight input nodes. The best performance is 0.05978 at epoch 147 with the lowest validation error.

**Figure 8:**
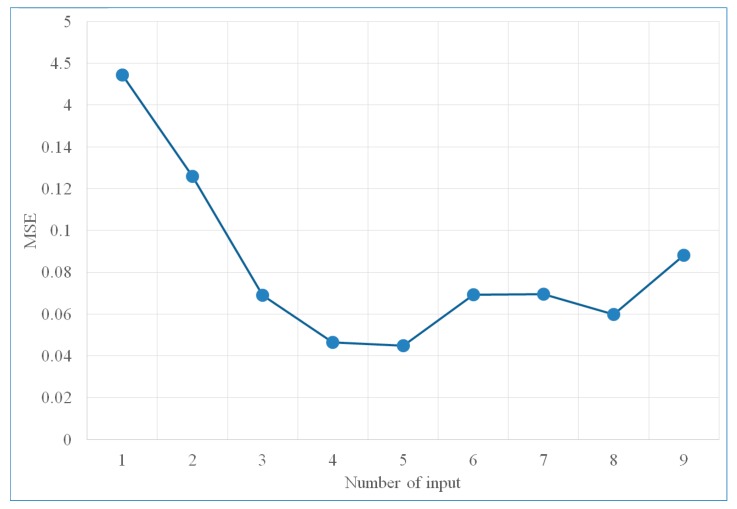
Training error of velocity prediction model with different input nodes.

We measure the MSE while varying the number of input nodes between 1 and 9. Table 1 and Figure 8 show the performance of velocity prediction model with different input nodes. The weights did not converge for the cases of 1 and 2 input nodes, where the maximum epoch was set as 1000. We can observe that the MSE increased when the number of input nodes is less than 4 and when it is more than 5. It means that if we use too few previous velocity data, we cannot get an accurate prediction. On the other hand, if we consider too much previous velocity data, the results may be impacted by the irrelevant information from a too long historical time period. When the number of nodes are 4 or 5, the proposed prediction model has a low MSE and thus give better prediction results. Thus, the number of nodes is selected as 4 or 5 in our method.

#### 4.2.2. Velocity Prediction Results

This experiment shows the complete prediction results. Based on the conclusions of the previous experiment, the prediction model with the lowest MSE has either 4 input nodes or 5 input nodes. In this experiment, we further estimate the error with mean absolute percentage error (MAPE), which is used to estimate the difference between actual values and predicted values in Equation (7):

(7) $$\mathrm{MAPE}=\frac{100}{n}\overset{n}{\underset{i=1}{\sum }}\left|\frac{A_{i}-P_{i}}{A_{i}}\right|$$

where *n* is the number of testing samples, *A_i_* is the actual velocity of *i*th testing sample, and *P_i_* is the predicted velocity of the *i*th testing sample.

**Figure 9:**
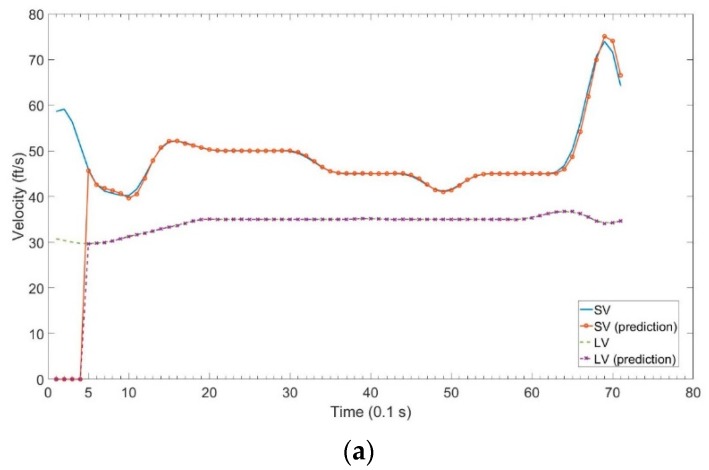

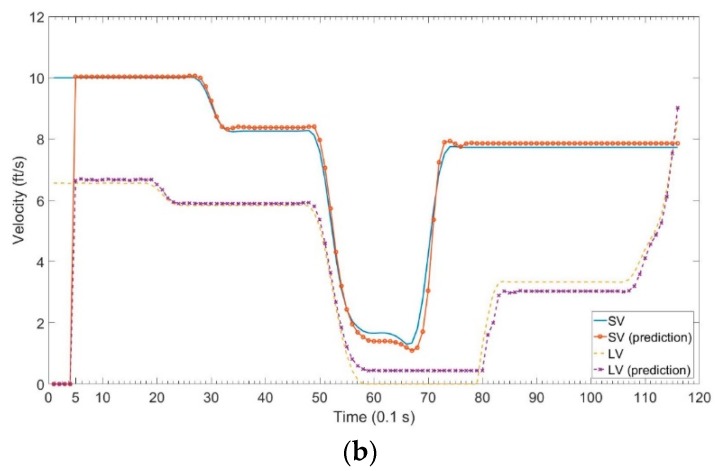
Comparison between actual velocity and predicted velocity with 4 inputs. (**a**) Comparison between actual velocity and predicted velocity in non-congested traffic scenario; (**b**) Comparison between actual velocity and predicted velocity in congested traffic scenario.

As a general standard of MAPE, a value of less than 10% indicates highly accurate prediction and that between 10% and 20% indicates excellent prediction [32]. Table 2 shows the MAPE for prediction horizons from 1 to 10 time slots. The MAPE of 4 input nodes is lower than that of 5 input nodes when the prediction horizon is larger than 7 time slots. Figure 9 shows the SV and LV prediction results compared to the actual velocity. We can see that the predicted velocity is zero initially because the input nodes are not enough to predict, then the predicted trend is very consistent.

#### 4.2.3. Collision Warning with Different Prediction Horizons

**Figure 10:**
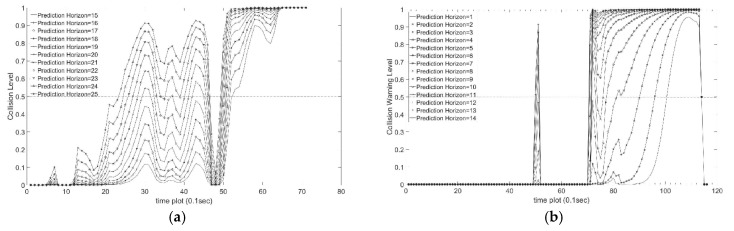
Variations in collision warning levels for different prediction horizons. (**a**) Non-congested Traffic Scenario; (**b**) Congested Traffic Scenario.

Variations in collision warning levels for different prediction horizons are shown in Figure 10 for both non-congested traffic scenario (15 to 25 time slots) and congested scenario (1 to 15 time slots). We can observe that the predicted collision warning levels are higher when the prediction horizon is larger. Since we take the maximum of all collision warning levels in the prediction horizon, the final resulting collision warning level monotonically increases with prediction horizon. The maximum function is used so that the final result indicates the level of collision risk within the prediction horizon. When the final collision warning level is above a given threshold, 0.5 here, then a rear-end collision warning alert is activated.

**Figure 11:**
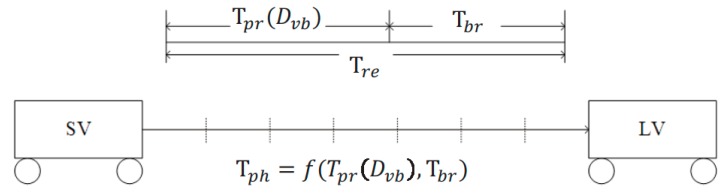
Collision warning timing analysis.

**Figure 12:**
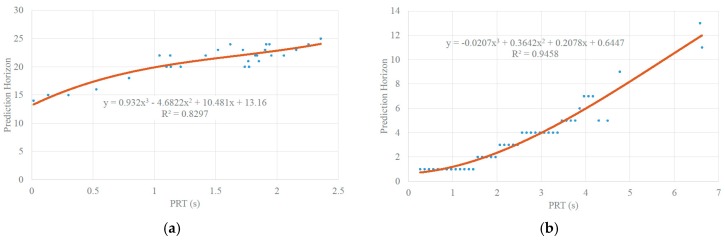
Relationships between prediction horizon and PRT. (**a**) Non-Congested Traffic Scenario; (**b**) Congested Traffic Scenario.

In the following, let us analyze the relation between the prediction horizon and PRT. As shown in Figure 11, if we want to avoid a predicted collision, an alert must be given at least *T_pr_* + *T_br_* time slots prior to the time of predicted collision. To avoid the collision in the time slot *T_br_*, a driver needs to react in the time slot *T_pr_*, brake with a deceleration *a_br_*, and decrease the SV’s speed to the same as the LV’s speed. It is suggested that *a_br_* is set to 6.56 ft/s^2^ (2 m/s^2^) to ensure ride comfort [7]. Then, *T_br_* can be computed as follows:

(8) $$T_{br}\left(t\right)=\frac{\Delta V}{a_{br}}=\frac{V_{SV}\left(t\right)-V_{LV}\left(T\right)}{a_{br}}$$

where Δ*V* is the difference between SV’s speed at time slot *t* and LV’s speed at time slot *T*. It means after *T_br_*, SV’s speed is the same as LV’s speed. *T* is set the 71st time slot in the non-congested traffic scenario, and the 115th time slot in the traffic congestion scenario, respectively, in which we assume a collision occurred. By assuming that the target (*LV*) becomes visible at time slot *t* under a given target visibility (in feet or meter), *T_pr_* can be calculated as follows:

(9) $$T_{pr}\left(t\right)=T_{re}-T_{br}\left(t\right)$$

where *T_re_*(*t*) is the time interval from time slot *t* to *T*. For each time slot and we experimented with different prediction horizons and found the prediction that gives the earliest collision alert for that time slot. These prediction horizons were mapped to *T_pr_*, which we assume is the PRT of SV. We then used a third order polynomial to fit the mapping data between prediction horizon and PRT of SV. As shown in Figure 12a, the resulting function of best fitting in the non-congested traffic scenario is as follows:

(10) $$T_{ph}=0.932T_{pr}^{3}-4.6822T_{pr}^{2}+10.481T_{pr}+13.16$$

where *T_pr_* is the SV’s PRT, and *T_ph_* is prediction horizon.

As shown in Figure 12b, the resulting function of best fitting in the congested traffic scenario is as follows:

(11) $$T_{ph}=0.0207T_{pr}^{3}+0.3642T_{pr}^{2}+0.2078T_{pr}+0.6447$$

Note that the above are just two examples of 3rd order polynomial fitting between PRT and prediction horizons. For different car following (SV and LV velocities and headway distances), we can always dynamically perform the fitting and decide on an appropriate prediction horizon so that car following by SV is safe and warnings of possible collision can be given much earlier in advance.

#### 4.2.4. Comparison with Different Methods

**Figure 13:**
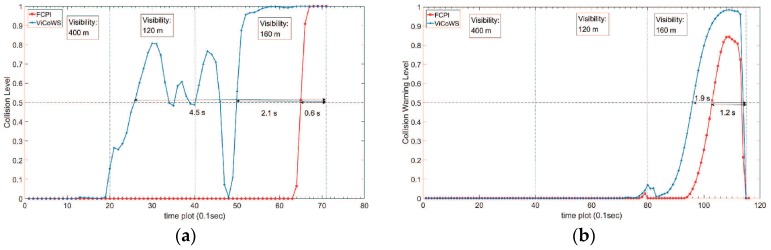
Comparison between ViCoWS and FCPI. (**a**) Non-Congested Traffic Scenario; (**b**) Congested Traffic Scenario.

For the non-congested traffic scenario, we experimented with different target visibility (*D_vb_*) conditions [19]. Between the time slots 1 to 19, the target visibility is 400 m. Between the time slots 20 to 39, the target visibility is 120 m due to a heavy fog. Between the time slots 40 to 71, the target visibility is 160 m due to a medium fog. The computed prediction horizons are shown in Table 3. Figure 13a shows the final result compared with the Forward Collision Probability Index (FCPI) method [29]. The FCPI method alerts SV in the 65th time slot, which gives 0.6s for the driver to react before collision. Our proposed ViCoWS method alerts SV between the 26th time slot to the 46th time slot, and between 50th time slot to 71st time slot. The collision warning level decreases between the 43th time slot to the 48th time slot because of the SV slowing down. The proposed ViCoWS method alerts the SV after the 50th time slot because the SV speeds up which leads to headway decreasing at a faster rate. If the SV did not brake between the 26th time slot to the 71st time slot, a collision will occur after 4.5 s. Further, if the SV did not brake in the 50th time slot to the 71st time slot, a collision will occur after 2.1 s.

As shown in Table 3, the warning time by ViCoWS (*T_ViCoWS_*) is always greater than the safe PRT (*T_PR_*) irrespective of the different visibilities, thus ViCoWS can safely warn drivers before it becomes dangerous. However, the warning time by FCPI (*T_FCPI_*) is safe only the normal visibility condition (400 m). The warning time of 0.6 before possible collision is much less than the PRT (2.0864 s under 120 m visibility and 1.6101 s under 160 m visibility).

For the congested traffic scenario, we also experimented with different target visibility (*D_vb_*) conditions. Between the time slots 1 to 39, the target visibility is 400 m. Between the time slots 40 to 79, the target visibility is 120 m due to a heavy fog. Between the time slots 80 to 115, the target visibility is 160 m due to a medium fog. The computed prediction horizons are shown in Table 4. Figure 13b shows the final result compared with FCPI, which alerts SV in the 103rd time slot, giving only 1.2 s for the driver to react before collision. Our proposed ViCoWS method alerts the SV between the 96th time slot to the 115th time slot. If the SV did not brake in time, a collision will occur after 1.9 s.

As shown in Table 4, under the 160 m visibility the safe warning time is *T_pr_* = 1.6101. The warning time by ViCoWS (*T_ViCoWS_*) is 1.9 s which is greater than the safe PRT (*T_pr_*) under the 160 m visibility, thus ViCoWS can safely warn drivers before it becomes dangerous. However, the warning time by FCPI (*T_FCPI_*) is 1.2 s before possible collision which is less than the safe PRT of 1.6101 s under 160 m visibility, thus FCPI is not able to cope with low visibility warnings.

It is shown that corresponding to different vehicle velocities, the ranges of prediction horizon must also be different so that collisions can be avoided. In the non-congested traffic scenario, the prediction horizons are in the range of 14 to 25 time slots. However, in the congested traffic scenario, the prediction horizons are in the range of 1 to 13 time slots. This shows that a vehicle with higher velocity should be warned earlier via a larger prediction horizon (more time slots) so that collisions can be avoided.

Further, more experiments were conducted to check the validity of the proposed ViCoWS method and to compare it with FCPI. As shown in Table 5, we experimented with 5 different sets of experiments, with a total of 567 datasets and 153 cases of target visibilities, the results show that ViCoWS gives early warnings in each case compared to FCPI. In Set A, the earliest warning by ViCoWS is at 4.6 s before collision which is safe (PRT should be at least 2.36 s), whereas FCPI warns by only 0.6 s before the collision, which is unsafe. The situations are similar for Sets B, C, D, and E. On average, ViCoWS is early by around 5.0 s which is 45.38% when compared to the full duration of car following before tentative collisions.

**Figure 14:**
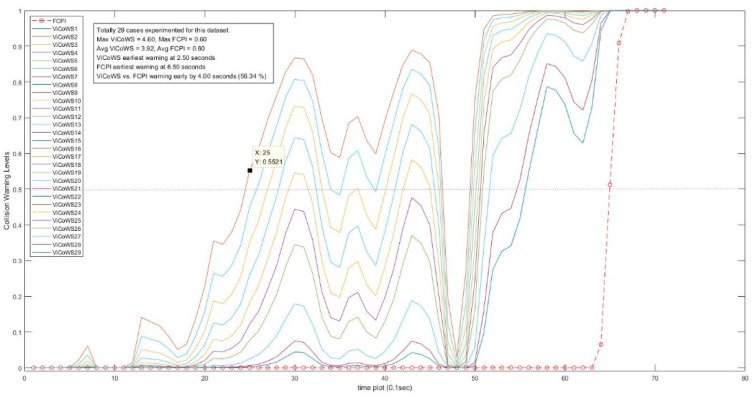
Comparison between ViCoWS and FCPI for 29 different visibilities (Set A).

**Figure 15:**
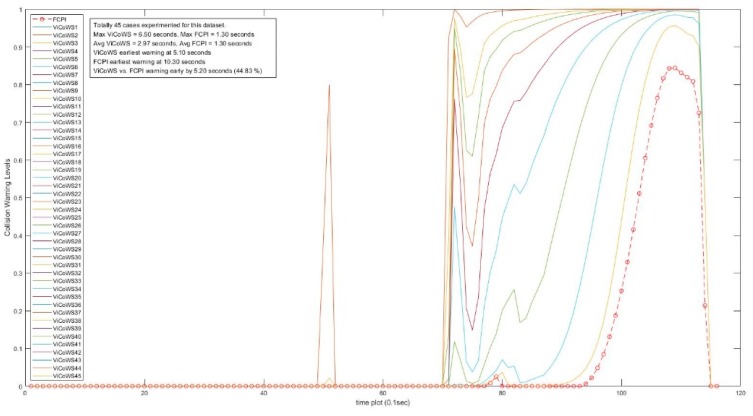
Comparison between ViCoWS and FCPI for 45 different visibilities (Set B).

**Figure 16:**
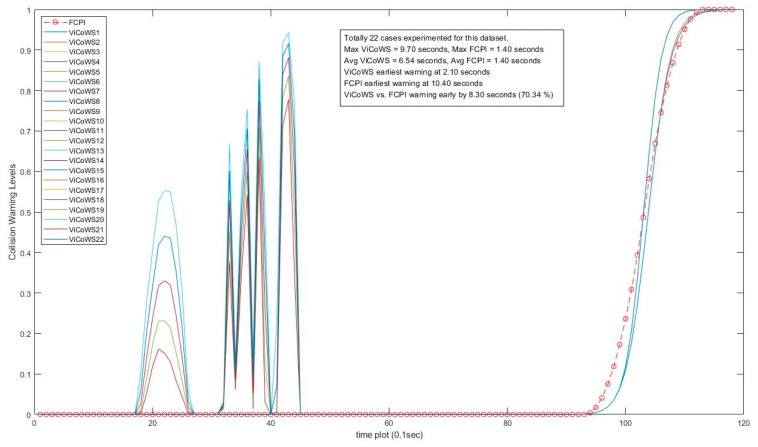
Comparison between ViCoWS and FCPI for 22 different visibilities (Set C).

**Figure 17:**
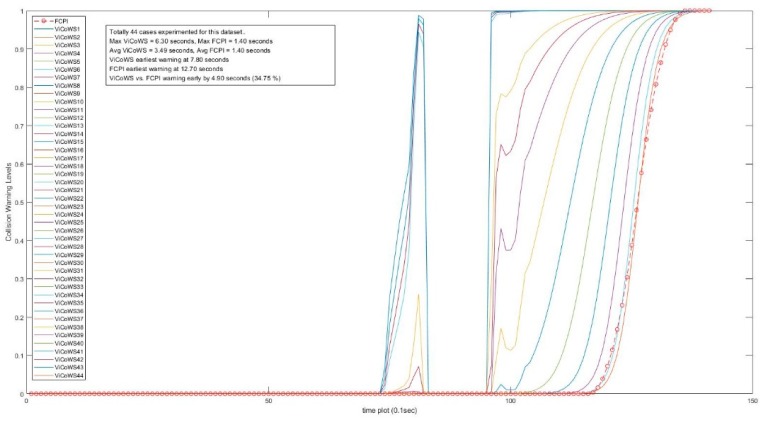
Comparison between ViCoWS and FCPI for 44 different visibilities (Set D).

**Figure 18:**
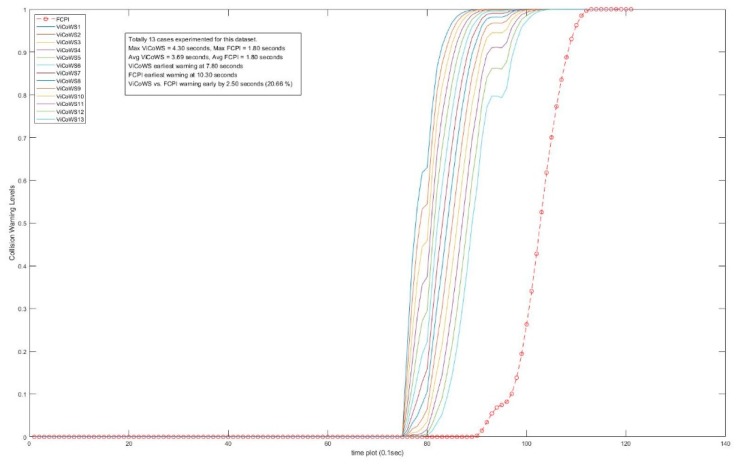
Comparison between ViCoWS and FCPI for 13 different visibilities (Set E).

From Table 5, we can conclude that ViCoWS is not only a valid method for giving early warnings of possible rear-end collisions in car following situations, but it is also much safer than the conventional FCPI method. The collision warning levels for different visibilities in the four datasets, including Set A, B, C, D, E experiments are illustrated in Figure 14, Figure 15, Figure 16, Figure 17 and Figure 18, respectively.

## 5. Conclusions

In this work, we proposed a Visibility-based Collision Warning System (ViCoWS) to predict future collision risks. In ViCoWS, the prediction horizons are adapted according to the different visibility conditions, such as foggy conditions. The velocity prediction model is based on back-propagation neural network (BPNN). The collision warning model is based on BPNN, which is used to predict the future collision warning levels for all time slots within the prediction horizon. ViCoWS alerts a driver when the collision warning level exceeds a threshold of 0.5. In non-congested traffic under low visibility conditions, ViCoWS can warn the driver by about 4.5 s prior to the collision (under visibility of 120 m) and 2.1 s prior to the collision (under visibility of 160 m). The FCPI method gives 0.6 s for the driver to react before collision. In congested traffic under low visibility conditions, ViCoWS can warn the driver by about 1.9 s prior to the collision, whereas the FCPI method gives 1.2 s for the driver to react before collision. In conclusion, irrespective of the traffic condition (non-congested or congested) and irrespective of the visibility conditions, the warning time by ViCoWS is always greater than the PRT thus it is safe; whereas that of FCPI fails to be safe in the case of congested traffic 160 m visibility. Further experiments all confirm the above claims.

In the future, we can further consider other deceleration values of SV when a driver needs to slow down to avoid a collision. We can also consider to learn drivers’ behavior, and change the threshold of collision warning level depending on different users. Moreover, with head-up display, ViCoWS can also be made to show the collision warning level in the form of green, yellow, or red light, which will be more intuitive. ViCoWS can also be applied to Cooperative Adaptive Cruise Control if it is combined with control methods that control vehicle velocity.

## Figures and Tables

**Table 1:** Training error of velocity prediction model with different input nodes.

# of Input Nodes	MSE	Epoch
1	4.362700	(not converge after 1000)
2	0.125990	(not converge after 1000)
3	0.068949	173
4	0.046333	79
5	0.044905	179
6	0.069214	111
7	0.069630	102
8	0.059780	147
9	0.088053	90

**Table 2:** Testing error of velocity prediction model.

	MAPE (%)									
# Input Nodes	Prediction Horizon (Time Slots)									
	1	2	3	4	5	6	7	8	9	10
4	0.32	1.08	2.16	3.39	4.61	5.73	6.57	7.26	8.03	8.90
5	0.26	0.90	1.88	3.00	4.23	5.42	6.75	8.06	9.15	9.96

**Table 3:** Prediction Horizons under different visibility for non-congested traffic scenario.

Time Slot	*D_vb_* (m)	*T_pr_* (s)	*T_ph_*	*T_ViCoWS_* (s)	*T_FCPI_* (s)
1 to 19	400	0.8397	19	No	No
20 to 39	120	2.0864	23	4.5~3.2	No *
40 to 71	160	1.6101	22	3.1~2.1	0.6 *

* Dangerous because Warning Time is less than Safe PRT.

**Table 4:** Prediction Horizons under different visibility for congested traffic scenario.

Time Slot	*D_vb_* (m)	*T_pr_* (s)	*T_ph_*	*T_ViCoWS_* (s)	*T_FCPI_* (s)
1 to 39	400	0.8397	1	No	No
40 to 79	120	2.0864	2	No	No
80 to 115	160	1.6101	2	1.9	1.2 *

* Dangerous because Warning Time is less than Safe PRT.

**Table 5:** Comparing ViCoWS and FCPI with more experiments.

Set	Dataset Size	No. of Visibility Cases	Target Visibility *D_vb_* (m)	Safe PRT *T_pr_* (s)	Prediction Horizon *T_ph_* (Time Slots)	Max *T_ViCoWS_* (s)	Max *T_FCPI_* (s)	Max Early (s)	Max Early (%)
A	71	29	106~444	2.36~0.79	25~18	4.6	0.6 *	4.0	56.34
B	116	45	39~516	6.48~0.74	13~1	6.5	1.3 *	5.2	44.83
C	118	22	37~44	7.11~5.83	25~16	9.7	1.4 *	8.3	70.34
D	141	44	50~488	5.08~0.76	23~3	6.3	1.4 *	4.9	34.75
E	121	13	221~515	1.24~0.74	25~20	4.3	1.8 *	2.5	20.66
**Total**	**567**	**153**			**Average Earlier by**			**5.0**	**45.38**

* Dangerous because Warning Time is less than Safe PRT.
